# Incidence of and Mortality from Type I Diabetes in Taiwan From 1999 through 2010: A Nationwide Cohort Study

**DOI:** 10.1371/journal.pone.0086172

**Published:** 2014-01-22

**Authors:** Wei-Hung Lin, Ming-Cheng Wang, Wei-Ming Wang, Deng-Chi Yang, Chen-Fuh Lam, Jun-Neng Roan, Chung-Yi Li

**Affiliations:** 1 Institute of Clinical Medicine, National Cheng-Kung University Hospital, National Cheng Kung University College of Medicine, Tainan, Taiwan; 2 Division of Nephrology, Department of Internal Medicine, National Cheng Kung University Hospital, College of Medicine, National Cheng Kung University, Tainan, Taiwan; 3 Institute of Clinical Pharmacy and Pharmaceutical Sciences, College of Medicine, National Cheng Kung University, Tainan, Taiwan; 4 Biostatistics Consulting Center, National Cheng Kung University Hospital, College of Medicine, National Cheng Kung University, Tainan, Taiwan; 5 Department of Anesthesiology, National Cheng Kung University Medical College, Tainan, Taiwan; 6 Division of Cardiovascular Surgery, Department of Surgery, National Cheng Kung University Hospital and College of Medicine, National Cheng Kung University, Tainan City, Taiwan; 7 Department and Graduate Institute of Public Health, College of Medicine, National Cheng Kung University, Tainan City, Taiwan; 8 Department of Public Health, College of Public Health, China Medical University, Taichung, Taiwan; Iran University of Medical Sciences, Islamic Republic of Iran

## Abstract

**Objective:**

To evaluate the secular trend in incidence of and mortality from Type 1 diabetes mellitus (T1DM) in Taiwan, 1999–2010.

**Methods:**

All 7,225 incident cases of T1DM were retrospectively retrieved from Taiwan's National Health Insurance Research Database from 1999 to 2010. Trend of bi-annual age- and sex-specific incidence rates of T1DM was calculated and tested with Poisson regression model. Standardized mortality ratios (SMRs) were calculated, using age-, sex-, and calendar years-specific mortality rates of the general population as the reference, to estimate the relative mortality risk of T1DM.

**Results:**

The number of male and female T1DM was 3,471 (48%) and 3,754 (52%), respectively. The annual number of incident T1DM increased from 543 in 1999 to 737 in 2010. The overall bi-annual incidence rate rose from 1999–00 to 2003–04 and mildly declined thereafter rose to 2009–10, with an insignificant trend (P = 0.489) over the study period. Regardless of gender, the higher age-specific incidence rate was noted in the younger groups (<30 years) and highest at <15 years. The incidence rates in younger groups were constantly higher in female population than in male one. The SMR from all causes was significantly increased at 3.00 (95% Confidence Interval (CI) 2.83–3.16) in patients with T1DM. The sex-specific SMR was 2.66 (95% CI 2.46–2.85) and 3.58 (95% CI 3.28–3.87) for male and female patients, respectively. For both sexes, the age-specific SMR peaked at 15–29 years.

**Conclusions:**

Among T1DM patients in Taiwan, there were significant increasing trends in males and female aged <15 years. We also noted a significantly increased overall and sex-specific SMR from all causes in patients with TIDM which suggests a need for improvements in treatment and care of patients with T1DM.

## Introduction

Type 1 diabetes mellitus (T1DM) has been reported to increase the risk of death [1], [2], mainly resulting from hyperglycemia, which is linked to a number of acute (e.g., diabetic ketoacidosis) and chronic (e.g., diabetic nephropathy and cardiovascular disease) complications [3]. Although treatment for T1DM improved evidently during the 1980s and 1990s with the advent of blood glucose self-monitoring, hemoglobin A1c testing, and use of angiotensin-converting-enzyme inhibitors/angiotensin II receptor blocker [4]–[6], T1DM complications still frequently lead to premature mortality [7]. Recent reports from Western Europe have shown a 3 to 4-fold higher long-term mortality rate (≧15 years follow-up) in T1DM, as compared to the general population [1], [8]. In the U.S., for example, the mortality rate of T1DM ranges from 5 to 7 times that of the general population [2], [7], and such increased risk of mortality was particularly notable for women and African Americans with T1DM. Risk of mortality from T1DM in Asian societies has rarely been reported in the literature.

A steady increase in the incidence of T1DM has been reported worldwide [9]. The incidence of this disease varies considerably among countries-e.g., it is lowest in China and Venezuela and highest in Finland and Sardinia [10]. However, long-term population-based data on T1DM incidence in the ethnic Chinese population is very limited [11], [12]. One national survey on whole diabetes found that T1DM was present in less than 1% of the diabetic population and the standardized incidence of T1DM remained constant over the recent 10 years in Taiwan [13]. No nationwide research focusing on T1DM mortality in Taiwan has been published. Using a large population-based T1DM cohort in Taiwan diagnosed between 1999 and 2010, we now investigated the long-term trends of incidence rate of T1DM in all sex and age stratifications after diagnosis and explored the overall as well as the age and sex-specific risk of mortality in patients with T1DM. Data analyzed in this study were derived from Taiwan's National Health Insurance Research Database (NHIRD) that provides a valid and nation-wide registration system for T1DM.

## Patients and Methods

### Data Sources

The National Health Insurance (NHI) service was launched in Taiwan on 1st March 1995. The coverage rate of the NHI from 2000 to 2007 had increased steadily from 96.1% to 98.6% [14]. The NHIRD, a large-scale computerized database, derived from this system by the Bureau of NHI and maintained by the National Health Research Institutes (NHRI), is provided to scientists in Taiwan for research purposes. Data in NHIRD that could be used to identify patients or care providers, including medical institutions and physicians, are scrambled before being sent to the National Health Research Institutes for database construction. Data are further scrambled before being released to each researcher. Therefore, individuals cannot be identified from the database.

In Taiwan, the certification of various catastrophic illnesses is subject to evaluation and review by the Bureau of NHI. Patients with catastrophic illness certificates (CICs) are eligible for exemption from insurance premiums and co-payments. Therefore, the CICs data are highly accurate and reliable [14]. The CICs will be terminated once the patients died. Access of this study to the NHIRD has been approved by the Review Committee of the NHRI, and conduct of this study was approved by the Institutional Review Board of National Cheng Kung University Hospital.

### Identification of newly-diagnosed type 1 diabetes mellitus

We searched the Taiwan NHIRD for the T1DM population from 1 January 1999 to 31 December 2010. (as per the International Classification of Diseases, 9th Revision, Clinical Modification, ICD-9-CM code 250). We considered the first ever registered CICs for T1DM as the newly-diagnosed T1DM patients, and excluded all CICs registered before 1999. T1DM is recognized as a catastrophic illness by the Taiwan NHI. Due to the fact that no co-payment is required for admission, emergency, and outpatient services, this certification is only applied when detailed and specific clinical data are met, e.g., regular insulin use with a history of diabetic ketoacidosis, a positive glucagon test, or the presence of glutamic-acid-decarboxylase (GAD) antibodies [13]. This CICs registration is a valid and reliable source of data for the T1DM retrieval. Besides, cases of newly-diagnosed T1DM retrieved from the CICs are considered complete [15].

### Validation

We validated the ICD-9-CM code for the identification of T1DM by analyzing the chart records of 60 patients who were randomly selected using the study method from the inpatient claims database in National Cheng Kung University Hospital, a 1143-bed tertiary medical center in southern Taiwan, between 2002 and 2010. The contents of this database were used for reimbursements and were similar to those of the NHIRD. T1DM was ascertained by the patients who had 3 or more outpatient diabetes diagnoses with insulin prescriptions, and a history of diabetic ketoacidosis, a positive glucagon test, or the presence of glutamic-acid-decarboxylase (GAD) antibodies [16], [17].

Among the randomly selected 60 patients coded with T1DM, 59 were confirmed by chart review, yielding a positive predictive value of 98.3%. Additionally, T1DM was listed in the principal diagnosis in 100.0% of the patients (data not shown in the table).

### Statistical Analysis

We first calculated the age- and sex-specific proportion of T1DM in Taiwan during the study period with the age stratifications of <15, 15–29, 30–44, 45–59, and 60 years or older. To obtain the reliable estimates of incidence rates, we calculated the age- and sex-specific biannual incidence rates of T1DM over the study period. The bi-annual incident rate of T1DM was calculated by dividing the number of incident T1DM cases by the averaged mid-year population of every two years. To examine the secular trend of T1DM incidence rate across the study period, we treated the calendar year as a continuous variable and testing the statistical significance of regression coefficient derived from the Poisson regression model that simultaneously included age, sex, and calendar year in the multivariable regression model. We also employed the 2000 WHO standard population as the reference to calculate the age- and sex-standardized incidence rate of overall T1DM bi-annually.

We selected age- and sex-matched control subjects by identifying from the national database with the age distribution of standard population and yearly mortality counting (i.e., population of Taiwan since 1999 to 2010) collected from Department of Statistics, Ministry of the Interior, Executive Yuan, Taiwan. Compared to the all-causes mortality rates of the general population of the same age and sex, we also calculated the age, sex, and calendar year standardized mortality ratio (SMR) of patients with T1DM. The 95% confidence intervals (CIs) of SMR were estimated using the Poisson distribution. The analyses were performed using SAS software (SAS Institute Inc., Cary, NC, USA). A P-value below 0.05 was considered statistically significant.

## Results

### Proportion and incidence of type 1 diabetes mellitus

**Figure 1:**
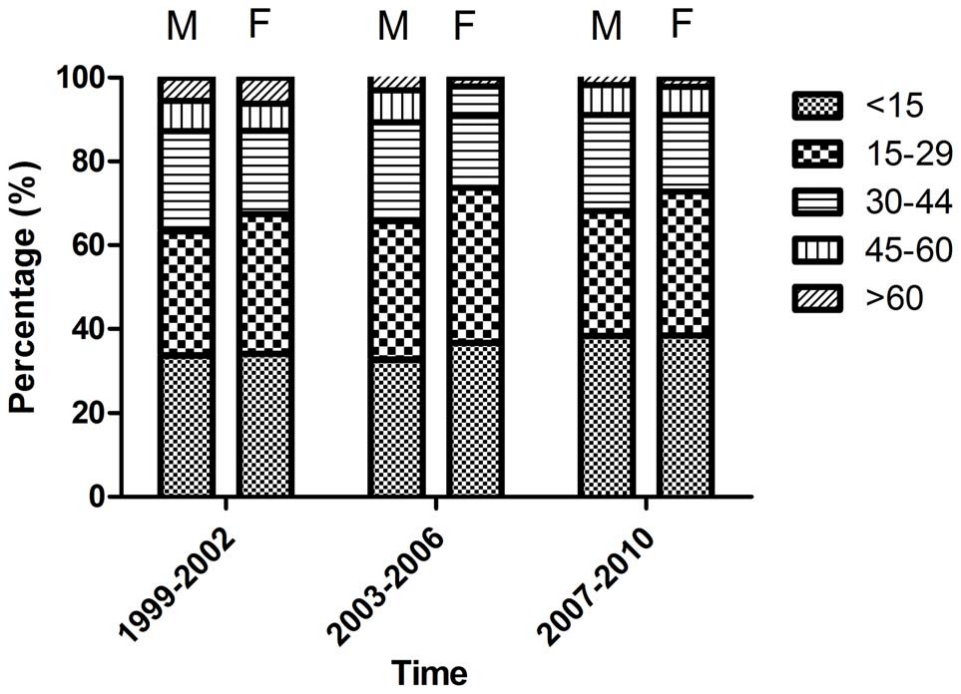
Age and sex specific proportions of newly-diagnosed type 1 diabetes in Taiwan from 1999 to 2010. (M: male, F: female).

Figure 1 shows the age and sex specific proportions of newly-diagnosed T1DM in Taiwan from 1999 to 2010. A total of 7,225 T1DM patients were identified from the CICs between 1999 and 2010 including 3,471 males and 3,754 females. The number of newly-diagnosed T1DM was increased from 1,133 in 1999–00 to 1,276 in 2009–10, and the number of female patients was generally greater than that of male patients. The majority of T1DM were diagnosed at ages of 30 years or less in both males and females. The age and sex-specific proportions of T1DM were generally constant over the study period. In male patients, the younger group (i.e., <30 years) accounted for about 60% of the patients, while the corresponding figure for female patients was even higher at 70%.

**Figure 2:**
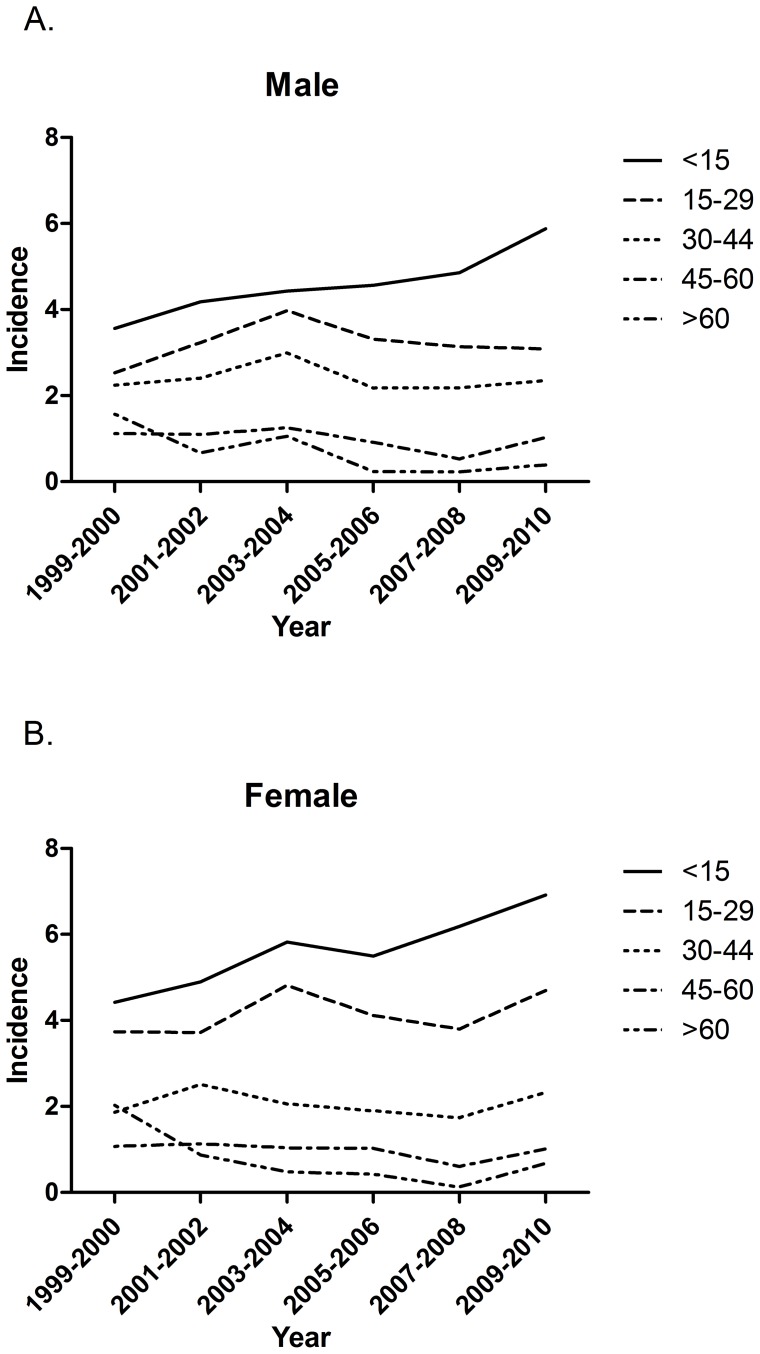
Trend of bi-annual age- and sex-specific incidence rate (per 100,000 inhabitants) of type 1 diabetes in Taiwan, 1999–2010. A. In male patients B. In female patients.

Table 1 shows the bi-annual overall as well as age- and sex-specific incidence rates of T1DM throughout 1999 to 2010. The standardized bi-annual overall incidence rate rose from 1999–00 (2.63 per 100,000) to 2003–04 (3.22 per 100,000) and then mildly declined thereafter rose to 2009–10 (3.34 per 100,000). Over the study period, the β value was −0.002 with an insignificant secular trend (P = 0.489). Regardless of gender, the highest age-specific incidence rate was noted in the younger group (<30 years) and especially highest at <15 years. Additionally, the incidence rate was constantly higher in females than in males in subjects aged <30 years during 1999 to 2010 (Figure 2). For both subjects, the mostly notable increase in incidence rate was noted for ages <15 yr in male (β = 0.043) and female (β = 0.041), respectively. Those aged 45–60 yr (β = −0.036) and ≧60 yr (β = −0.176) in male subjects and those aged ≧60 yr (β = −0.169) in female subjects showed the greater decreases.

**Table 1:** Bi-annual sex- and age-specific incidence rate (per 100,000 inhabitants) of type 1 diabetes in Taiwan, 1999–2010.

	1999–00	2001–02	2003–04	2005–06	2007–08	2009–10	β	P for trend
**Male**								
<15 yr	3.56	4.19	4.43	4.56	4.86	5.88	0.043	<0.001*
15–29 yr	2.53	3.23	3.97	3.31	3.14	3.08	0.009	0.322
30–44 yr	2.24	2.40	2.99	2.18	2.18	2.35	−0.006	0.590
45–60 yr	1.12	1.10	1.25	0.92	0.53	1.02	−0.036	0.049*
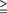 60 yr	1.57	0.67	1.06	0.23	0.23	0.39	−0.176	<0.001*
**Female**								
<15 yr	4.42	4.89	5.82	5.50	6.19	6.92	0.041	<0.001*
15–29 yr	3.74	3.72	4.81	4.12	3.80	4.70	0.015	0.063
30–44 yr	1.87	2.51	2.06	1.89	1.74	2.32	−0.001	0.916
45–60 yr	1.07	1.13	1.03	1.02	0.60	1.01	−0.027	0.142
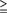 60 yr	2.03	0.87	0.48	0.42	0.12	0.67	−0.169	<0.001*
**Total**	2.63	2.83	3.22	2.85	2.81	3.34	−0.002	0.489

* P for linear trend test <0.05.

### Mortality of type 1 diabetes mellitus

The all causes SMR in patients with T1DM was significantly increased at 3.00 (95% CI, 2.83–3.16). The sex-specific SMR for men and women was also significantly increased at 2.66 (95% CI 2.46–2.85) and 3.58 (95% CI 3.28–3.87) (Table 2). Among the age stratifications, the SMR peaked when age at onset was 15–29 years for both male (5.97, 95% CI 4.99–6.95) and female (12.91, 95% CI 10.96–14.86) subjects. The SMR was significantly increased at all age stratifications in females, but age at onset <60 years in male subjects.

**Table 2:** Standardized mortality ratio of type 1 diabetes in Taiwan according to sex and age at onset, 1999–2010.

	Observedno. of deaths	Expected no. of deaths	SMR	95% CI
**All patients**	333	111.08	3.00	**2.83**–**3.16**
Male	186	69.99	2.66	**2.46**–**2.85**
Female	147	41.08	3.58	**3.28**–**3.87**
**Age at onset, male**				
<15 yr	11	2.39	4.60	**3.21**–**5.98**
15–29 yr	37	6.20	5.97	**4.99**–**6.95**
30–44 yr	75	14.15	5.30	**4.69**–**5.91**
45–60 yr	30	13.37	2.24	**1.83**–**2.65**
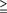 60 yr	33	35.33	0.93	0.77–1.10
**Age at onset, female**				
<15 yr	10	2.30	4.34	**2.97**–**5.71**
15–29 yr	44	3.41	12.91	**10.96**–**14.86**
30–44 yr	34	4.29	7.92	**6.56**–**9.27**
45–60 yr	25	5.49	4.55	**3.64**–**5.46**
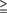 60 yr	34	26.56	1.28	**1.06**–**1.50**

Abbreviations: CI, confidence interval; SMR, standardized mortality rate.

Table 3 shows the SMR of T1DM according to the duration since diagnosis of T1DM. Among the age stratifications after T1DM onset, the SMR were significantly increased at all following time for both gender after T1DM diagnosis. The SMR for male was increased from 1.78 (95% CI 1.35–4.63) in the first year to 2.84 (95% CI 2.57–3.52) for the period of <5 yr then mildly declined to 2.66 (95% CI 2.46–2.85) for the period of <12 yr. The SMR for female was highest from 5.00 (95% CI 4.02–9.09) then declined to 3.58 (95% CI 3.28–3.87) for the period of <12 yr.

**Table 3:** Standardized mortality ratio of type 1 diabetes in Taiwan according to duration of disease by gender, 1999–2010.

	Observedno. of deaths	Expected no. of deaths	SMR	95% CI
**Duration (yrs), male**				
<1	17	9.53	1.78	**1.35**–**4.63**
<3	69	26.02	2.65	**2.33**–**3.69**
<5	113	39.76	2.84	**2.57**–**3.52**
<12	186	69.99	2.66	**2.46**–**2.85**
**Duration (yrs), female**				
<1	26	5.20	5.00	**4.02**–**9.09**
<3	56	14.08	3.98	**3.45**–**5.49**
<5	89	21.87	4.07	**3.64**–**5.04**
<12	147	41.08	3.58	**3.28**–**3.87**

Abbreviations: CI, confidence interval; SMR, standardized mortality rate.

To assess the duration specific SMR in children and adults separately, we further presented age stratified results (Table 4). Among the group of <15 yr, the SMR was 3.65 (95% CI 1.83–5.48) in the first year and slightly went down to 2.35 (95% CI 1.46–3.24) for the period of <3 yr, then rose to 4.47 (95% CI 3.50–5.45) for the period of <12 yr. Among the group of ≧15 yr, the SMR rose from 2.86 (95% CI 2.40–3.32) in the first year to 3.31 (95% CI 3.07–3.55) for the period of <5 yr then declined to 2.87 (95% CI 2.71–3.03) for the period of <12 yr.

**Table 4:** Standardized mortality ratio of type 1 diabetes in Taiwan according to duration of disease by <15 yr and ≧15 yr, 1999–2010.

	Observedno. of deaths	Expected no. of deaths	SMR	95% CI
**Duration (yrs), <15 yr**				
<1	4	1.10	3.65	**1.83**–**5.48**
<3	7	2.98	2.35	**1.46**–**3.24**
<5	13	4.47	2.91	**2.10**–**3.72**
<12	21	4.70	4.47	**3.50**–**5.45**
**Duration (yrs), 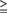 15 yr**				
<1	39	13.64	2.86	**2.40**–**3.32**
<3	118	37.12	3.18	**2.89**–**3.47**
<5	189	57.16	3.31	**3.07**–**3.55**
<12	312	108.81	2.87	**2.71**–**3.03**

Abbreviations: CI, confidence interval; SMR, standardized mortality rate.

## Discussion

This is the largest population-based T1DM cohort study with at least 12 years of follow-up in Taiwan. We noted that the incidence rate of T1DM was higher in women, especially in the younger group (age at onset <30 years) and was highest when age at onset <15 years both in both sexes. Additionally, the all causes SMR was significantly increased in female patients of all age stratifications. The age-specific SMR was peaking when age at onset was 15–29 years for both sexes. Moreover, the significantly increased SMR was also noted for both gender in the variable following time especially highest for female after disease diagnosis less than one year. From our study we also found that the trend of SMR is different if the diagnosis of T1DM is in childhood (<15 yr) or in adulthood (≧15 yr).

It has been well-documented that the incidence of T1DM is higher among Caucasoid populations than among Mongoloids and Blacks. The distribution of ethnic diversity shows the importance of the differential genetic susceptibility among populations. Within ethnic groups, however, there are also geographical differences in incidence largely explained by the admixture between racial groups and possible environmental exposures [18]. The incidence of T1DM possibly has been underestimated in earlier studies because of incomplete case ascertainment and death from undiagnosed diabetes. Compared with the Chinese population study in Shanghai [19], [20], our group may provide more reliable and updated estimates of incidence and mortality rates in patients with ethnic Chinese T1DM patients due to certain methodological strengths including a much larger and representative study cohort. A steady increase in the incidence of T1DM has been reported worldwide [9]. From our study, we found an upward trend in the incidence of T1DM in younger age-groups (<15 yr), which is similar to the findings of other countries, but a downward trend in older age-groups (≧60 yr). There are several possible reasons that may explain the downward trend among old ages in our study. Free health check-ups have been available for adults aged 40 years or older since 1995 when Taiwan's National Health Insurance program was lunched. A diagnostic protocol for diabetes was also proposed using fasting plasma glucose and hemoglobin A1c levels as criteria. The American Diabetes Association also lowered the fasting plasma glucose level (i.e.,126 mg/dL) in determining diabetes in 1997. Since we enrolled the study patients registered in 1999 and thereafter, therefore, the elderly T1D registered in earlier years might include some patients who had delayed diagnosis.

Studies of patients with T1DM have shown relative mortality peaking in young adults. For example, both the Pittsburgh and Denmark studies showed that relative mortality was highest in the 30–39 ages [21], [22]. The New Zealand and UK studies showed that the highest relative mortality occurred in the 0–29 age group [23], [24]. Our study also revealed a higher relative risk of mortality noted in the younger (age at onset <45 years) group and the highest when age at onset was 15–29 years, which was compatible with findings of the previous studies [21]–[24]. The higher relative risk of death for T1DM patients diagnosed during adolescence and young adults may reflect that these patients are essentially out of their parents' control at the time of diagnosis, increasing the risk of diabetic ketoacidosis (DKA) or poor overall diabetes management [25]. Besides, the severity of T1DM itself would be different across age groups; that is, patients who have severe conditions or rapidly progressive T1DM could be detected in younger age and subsequently have a higher mortality risk.

Many studies have shown that there are no gender differences in mortality associated with T1DM, either in terms of death rates or relative risk of mortality [26]–[28]. In contrast, the risk of mortality relative to the New Zealand general population was higher at all ages in female T1DM than in males except in the 40–49 age group at onset, and the largest difference was noted in the 0–29 age group at onset [23]. Results from the British Diabetic Association Cohort Study also showed a higher mortality in diabetic females than in males at all ages at onset compared to the general population [24]. Finland was the country with the world's highest incidence of T1DM and had many related researches [10], [29]. In a population based nationwide cohort study conducted in Japan and Finland, women had a higher relative mortality in both country but men had a higher absolute mortality in the Finnish cohort. These previous findings suggested a greater effect of T1DM on mortality in female population than in males [10], [29].These previous findings are generally further supported by our study findings, showing a significantly higher SMR among female patients. One possible explanation for a higher SMR in female T1DM patients may be related to our previous findings that although the overall incidence of DKA linearly decreased between 1997 and 2005 in Taiwan, significantly sharp rising trends were still observed in female diabetic patients aged <35 years [30]. It has been reported that, to have a better body image, girls with diabetes often omit insulin injections to avoid weight gain [31]. Rodin et al also reported that young female patients with T1DM tend to have a relatively high prevalence of eating disorders, which might contribute to impaired metabolic control, and consequently result in hyperglycemia and DKA [32]. More aggressive diabetic care programs aimed at young female patients should be considered to reduce this emergency and possibly fatal diabetic complications. Since women usually have lower absolute rates of mortality than men at these ages in Taiwan, clinicians must be acutely aware of this differential and be aggressive in diabetes management.

The Pittsburgh Epidemiology of Diabetes Complications (EDC) data confirmed the importance of renal disease, including persistent microalbuminuria, as a marker of mortality risk and suggested that T1DM patients without renal disease achieve long-term survival comparable to the general population [33]. Taiwan was a country with a very high prevalence of end stage renal disease (ESRD) and diabetes was the leading cause [34]. Whether or to what extent the ESRD may have explained the increased risk of mortality from Taiwanese T1DM population warrants further investigations.

For males, the SMR gradually increased with the increasing duration of T1DM. But for females, the highest SMR was noted during the first year, and decreased after 5 years. We also found that the SMR went down after the first year, and then increased with increasing duration of follow-up in children aged <15 yr. Patients with T1DM have been found to experience various acute and chronic complications. DKA has been reported to be the leading cause of early mortality in T1DM, which may occur long before the peak incidence of chronic complications of diabetes [8], [35]–[37]. The EURODIAB mortality study reported that as many as 35% of the deaths in children diagnosed after 1989 were due to acute complications with DKA [35]. Although the aetiology is not fully clear, it is found that the incidence of T1DM is relative low, but the frequency of DKA in T1DM is very high (65%) in Taiwan [38], [39]. A previous Taiwanese study [30] reported a significant rising trend of DKA incidence in female diabetic patients aged <15 years between 1997 and 2005, but such rising trend was not observed in male patients of the same age, which may largely contribute to our finding that a high SMR in the first years of T1DM diagnosis in females, especially in the first year. It is therefore suggested that more aggressive diabetic cares aiming at young female patients are warranted. Physicians should also be more alert of the increased risk of death, especially from DKA, in children or younger adults diagnosed with T1DM.

The strengths of this work included its nationwide population-based cohort design, relative long duration of follow-up (up to 12 years), and the large number of incident cases of T1DM, which allowed us to calculate the incidence and mortality according to various sex and age stratifications. In addition, the study used the registration system of catastrophic diseases in Taiwan, which provides a good validation of disease diagnosis. Besides, based on the universal coverage system, all T1DM patients were enrolled in the study and a nationally representative sample can be assumed in our study. Findings from our study may provide the updated information on T1DM incidence and mortality in Asian populations. Despite the above strengths, certain limitations involved in our study should be noticed. First, the claim data do not cover laboratory data and clinical details specific to T1DM, which made it impossible to determine the severity of T1DM. Second, there is no information on the underlying cause of death in the NHI claims, thus, we were unable to estimate the cause-specific risk of mortality in T1DM.

## Conclusions

In summary, the incidence rate of T1DM was highest in the young-aged group (age at onset <15 years) in both women and men in Taiwan, and the difference in incidence rate between sexes was also greatest in this young group. We also noted an excess mortality in Taiwan's T1DM patient population of both sexes, and of many age stratifications. Targeted strategies aimed at reducing mortality among individuals with T1DM must be considered from both clinical and health policy perspectives.
